# Transcriptional regulation of abscisic acid biosynthesis and signal transduction, and anthocyanin biosynthesis in ‘Bluecrop’ highbush blueberry fruit during ripening

**DOI:** 10.1371/journal.pone.0220015

**Published:** 2019-07-18

**Authors:** Sun Woo Chung, Duk Jun Yu, Hee Duk Oh, Jong Hwa Ahn, Jin Hoe Huh, Hee Jae Lee

**Affiliations:** 1 Department of Plant Science, Seoul National University, Seoul, Republic of Korea; 2 Research Institute of Agriculture and Life Sciences, Seoul National University, Seoul, Republic of Korea; Zhejiang University, CHINA

## Abstract

Highbush blueberry (*Vaccinium corymbosum*) fruit accumulate high levels of anthocyanins during ripening, which might be controlled by abscisic acid (ABA), a signal molecule in non-climacteric fruits. For an integrated view of the ripening process from ABA to anthocyanin biosynthesis, we analyzed the transcriptomes of ‘Bluecrop’ highbush blueberry fruit using RNA-Seq at three ripening stages, categorized based on fruit skin coloration: pale green at ca. 30 days after full bloom (DAFB), reddish purple at ca. 40 DAFB, and dark purple at ca. 50 DAFB. Mapping the trimmed reads against the reference sequences yielded 25,766 transcripts. Of these, 143 transcripts were annotated to encode five ABA biosynthesis enzymes, four ABA signal transduction regulators, four ABA-responsive transcription factors, and 12 anthocyanin biosynthesis enzymes. The analysis of differentially expressed genes between the ripening stages revealed that 11 transcripts, including those encoding nine-*cis*-epoxycarotenoid dioxygenase, SQUAMOSA-class MADS box transcription factor, and flavonoid 3′,5′-hydroxylase, were significantly up-regulated throughout the entire ripening stages. In fruit treated with 1 g L^−1^ ABA, at least nine transcripts of these 11 transcripts as well as one transcript encoding flavonoid 3′-hydroxylase were up-regulated, presumably promoting anthocyanin accumulation and fruit skin coloration. These results will provide fundamental information demonstrating that ABA biosynthesis and signal transduction, and anthocyanin biosynthesis are closely associated with anthocyanin accumulation and skin coloration in highbush blueberry fruit during ripening.

## Introduction

Climacteric fruits such as apple, banana, and tomato, generate a burst of ethylene at the onset of ripening [1–3]. The burst of ethylene accelerates ripening of climacteric fruits. These changes act as a signal of the initiation of ripening in all climacteric fruits. Ripening of climacteric fruits is also stimulated by exogenous ethylene. In contrast, non-climacteric fruits, including strawberry, grape, and blueberry, do not show a dramatic change in ethylene content, and are not affected by exogenous ethylene [4–6], although some such fruits have ethylene receptors [7]. However, ripening of non-climacteric fruits in association with hormonal regulation remains poorly understood.

Evidence that the ripening of non-climacteric fruits is associated with abscisic acid (ABA) has been accumulated. Fruit coloration during ripening is promoted by ABA application in many non-climacteric fruits, including blueberry [8], grape [9–11], strawberry [5, 12], and sweet cherry [13, 14]. Genes involved in ABA biosynthesis and signal transduction have been reported to be regulated during ripening as those in anthocyanin biosynthesis and furthermore their regulations were enhanced by ABA application. For example, *ß-carotene 3-hydroxylase* (*BCH*) [15] and *nine-cis-epoxycarotenoid dioxygenases* (*NCED*s) [9, 16] involved in ABA biosynthesis and *pyrabactin resistance/pyrabactin resistance-like/regulatory components of ABA receptors* (*PYR*/*PYL/RCAR*) involved in ABA signal transduction were up-regulated during ripening. Conversely, silencing *NCED*s resulted in colorless phenotypes in strawberry [12], sweet cherry [14], and bilberry fruits [17]. The *PYR*-silenced strawberry fruit did not undergo skin coloration regardless of ABA application [5]. These findings suggest that ABA biosynthesis and signal transduction are closely associated with anthocyanin biosynthesis during ripening.

Blueberry fruit accumulate high level of anthocyanins during ripening, leading to a highly noticeable coloration [8, 16]. The coloration with anthocyanin accumulation makes blueberry fruit suitable for studies of ripening. As the blueberry fruit undergo ripening, their skin color changes from pale green to dark blue or purple according to the accumulations of the individual anthocyanins derived from a particular anthocyanidin type [8, 16, 18, 19]. Correlation of fruit skin coloration and anthocyanin accumulation during ripening has often been demonstrated in blueberry fruit [8, 18].

Transcriptome analysis using RNA-Seq has widely been applied to explain various cellular metabolisms [20–22]. Although transcripts encoding the enzymes involved in anthocyanin biosynthesis have been sequenced in blueberry fruit [16, 23–26], the transcript expressions regarding ABA and anthocyanin biosynthesis have not been investigated for explaining fruit coloration during ripening.

In this study, the transcriptomes of ‘Bluecrop’ highbush blueberry (*Vaccinium corymbosum*) fruit were analyzed using RNA-Seq to obtain an integrated view of the ripening process from ABA to anthocyanin biosynthesis. We also characterized the effects of exogenous ABA on anthocyanin accumulation and its regulatory transcript expression.

## Materials and methods

### Plant materials

**Fig 1:**
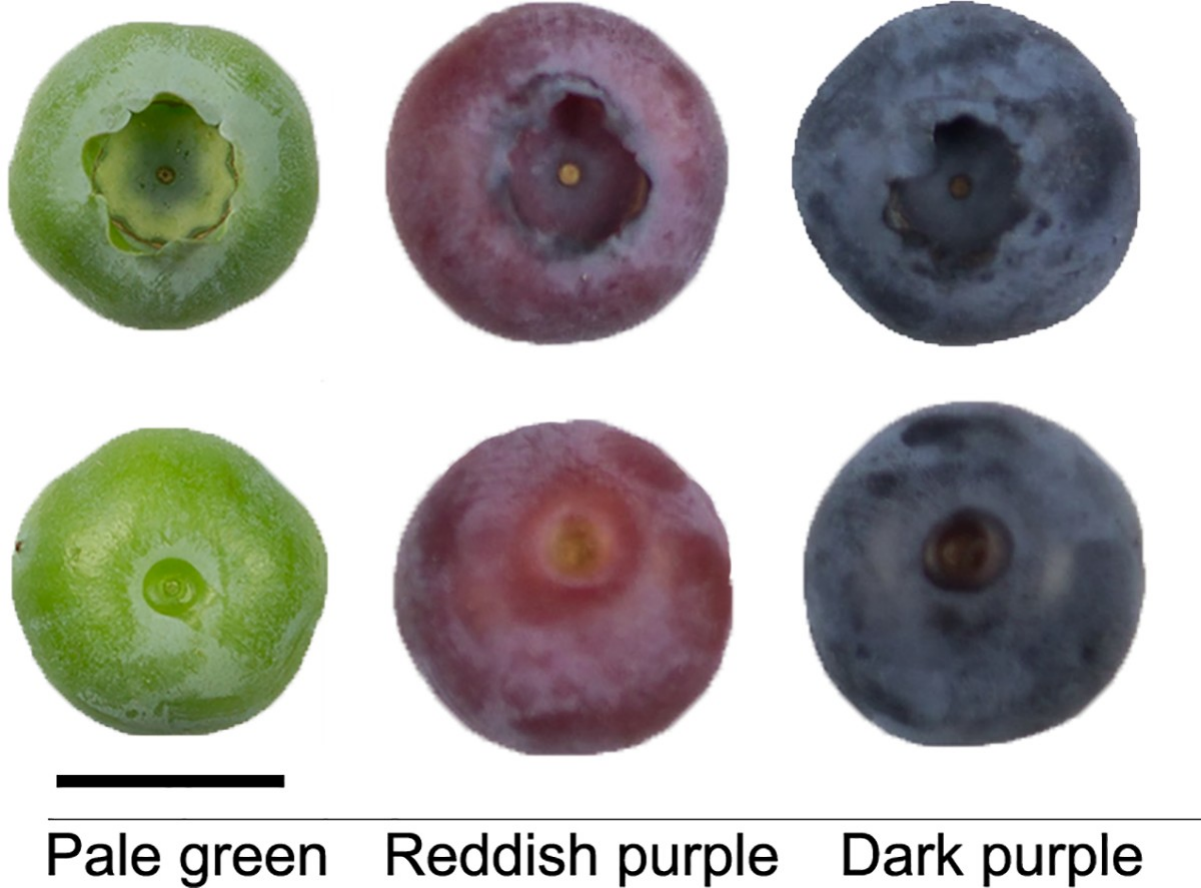
**Calyx end (top) and stem end (bottom) of ‘Bluecrop’ highbush blueberry fruit at three ripening stages based on skin coloration: pale green at ca. 30 days after full bloom (DAFB), reddish purple at ca. 40 DAFB, and dark purple at ca. 50 DAFB.** Bar = 1 cm.

Ten-year-old ‘Bluecrop’ highbush blueberry shrubs were grown in the experimental orchard of Seoul National University, Suwon (37° 17’ N, 127° 00’ E), Republic of Korea. Fruit were categorized into three ripening stages based on their skin coloration: (1) pale green at ca. 30 days after full bloom (DAFB), (2) reddish purple at ca. 40 DAFB, and (3) dark purple at ca. 50 DAFB (Fig 1). Ninety fruit at each stage were harvested from three shrubs during ripening to provide three replicates with 30 fruit each in the sampling design. All harvested fruit were immediately frozen in liquid nitrogen and stored at –80°C until use for the transcriptome analysis.

### ABA treatments

Fruit clusters attached to the shrubs were dipped into a solution containing 1 g L^–1^ (±) ABA (Sigma-Aldrich, St. Louis, MO, USA) for 1 min at pale green stage, according to the methods of Jeong et al. [27]. The ABA concentration was chosen based on the results of our previous study [8], and the ABA solution was prepared with 5% ethanol containing 0.1% (v/v) Tween 80. All treatments were conducted after sunset to avoid the photodegradation of ABA [28]. Control fruit were treated with 5% ethanol containing 0.1% Tween 80 without ABA. These experiments were employed in a randomized complete block design with three replicates consisting of five shrubs each. Fifty fruit were randomly sampled from each replicate block at 5 days after treatment (DAT) when the fruit skin color began to change. All harvested fruit were immediately frozen in liquid nitrogen and stored at –80°C until use for the quantification of individual anthocyanins and the expression profiling of their related transcripts.

### Determination of fruit color

Fruit skin colors were measured using a spectrophotometer (CM-2500d; Minolta Co., Osaka, Japan) and described by the CIE L*, a*, and b* color space coordinates [29]. The L* value represents the lightness of colors, with a range of 0 to 100 (0, black; 100, white). The a* value is negative for green and positive for red. The b* value is negative for blue and positive for yellow. For each fruit, the values were measured at three different points along the fruit equator.

### Determination of individual anthocyanin contents

Anthocyanins were extracted according to the method described by Gavrilova et al. [30], with some modifications. Approximately 5 g of ground fruit tissues was added to 10 mL of a solution containing acetone:acetic acid (99:1, v/v). The homogenates were sonicated for 15 min and centrifuged at 1,900 × *g* for 15 min. The supernatants were evaporated until dry using a rotary evaporator (EYELA N-1000S-W; Tokyo Rikakikai Co., Tokyo, Japan) at 37°C, then completely redissolved in 10 mL of 20% methanol and filtered through a PTFE filter with a pore size of 0.45 μm (Whatman Inc., Florham Park, NJ, USA).

Individual anthocyanin contents were determined using a high-performance liquid chromatography (HPLC)-diode array detector system (Dionex Ultimate 3000; Thermo Fisher Scientific, Waltham, MA, USA) equipped with a VDSpher PUR C-18 column (4.6 mm × 150 mm, 3.5 μm; VDS Optilab, Berlin, Germany). Anthocyanins were eluted using a gradient of mobile phase A (aqueous 5% [v/v] formic acid) and mobile phase B (5% [v/v] formic acid in acetonitrile) in the following sequence: 0–30 min, 5–45% B; 30–35 min, 45% B; 35–36 min, 45–5% B; and 36–40 min, 5% B. The flow rate was 0.8 mL min^–1^, and detections were made at 520 nm. Cyanidin, delphinidin, peonidin, and petunidin 3-*O*-glucosides (Sigma-Aldrich), and malvidin and pelargonidin 3-*O*-glucosides (Polyphenols AS, Sandnes, Norway) were used as standards.

### RNA extraction

Total RNA was extracted from fruit at each stage as described by Jaakola et al. [31], with slight modifications. Extraction buffer (2% hexadecyltrimethylammonium bromide, 2% polyvinylpyrrolidone, 100 mM Tris-HCl [pH 8.0], 25 mM EDTA [pH 8.0], 2.0 M NaCl, and 2% β-mercaptoethanol) was heated to 65°C and then 900 μL of the extraction buffer was transferred to a 2-mL microfuge tube containing 100 mg of powdered fruit tissues and incubated at 65°C for 10 min. An equal volume of chloroform:isoamyl alcohol (24:1, v/v) was added, vortexed for 5 s, and centrifuged at 10,000 × *g* at 4°C for 10 min. The supernatant of 750 μL was recovered and mixed with an equal volume of chloroform:isoamyl alcohol. Following the centrifugation as above, the supernatant of 600 μL was transferred to a new 2-mL tube, and an equal volume of 6 M LiCl solution was added. The mixture was incubated on ice for 30 min and centrifuged at 21,000 × *g* at 4°C for 20 min to precipitate the RNA. The pellet was resuspended in 500 μL of preheated (65°C) SSTE buffer (0.5% sodium lauryl sulfate, 1 M NaCl, 1 M Tris-HCl [pH 8.0], and 10 mM EDTA [pH 8.0]) while gentle shaking. An equal volume of chloroform:isoamyl alcohol was added, and the mixture was centrifuged at 21,000 × *g* at 4°C for 10 min, then dried and resuspended in 20 μL diethyl pyrocarbonate-treated water. Finally, the solution was heated at 65°C for 5 min to completely dissolve the RNA. The quality of the extracted RNA samples was assessed using a NanoDrop ND1000 (Thermo Fisher Scientific), following the confirmation of the RNA integrity using an Agilent 2100 Bioanalyzer (Agilent Technologies, Waldbronn, Germany).

### RNA-Seq and sequence processing

Nine cDNA libraries were constructed for fruit at pale green, reddish purple, and dark purple stages from three replicates with 30 fruit each, using a TruSeq small RNA library preparation kit (Illumina, San Diego, CA, USA), and sequenced using an Illumina HiSeq 2000 system. The quality of the data produced was confirmed using the R package fastqcr (version 0.1.2). Adapters and low-quality reads, including short reads (< 36 bp) and reads with a Phred score Q ≤ 20, were removed from the raw data using the R package QuasR (version 1.22.1). The trimmed reads were mapped to the reference highbush blueberry transcriptome (*V*. *corymbosum* RefTrans V1) from the Genome Database for *Vaccinium* using Cufflinks (version 2.2.1). The RNA-Seq data were deposited to the National Center for Biotechnology Information (NCBI) (accession No. PRJNA533973).

### Gene ontology (GO) annotation and identification of differentially expressed genes (DEGs)

GO assignments were made to the mapped reads using InterProScan at the European Bioinformatics Institute through Blast2GO. The obtained GO terms were classified and plotted using WEGO (version 2.0).

To identify the DEGs, the mapped transcripts were functionally annotated using the KEGG database, and the expression levels of the transcripts were calculated as fragments per kilobase of transcript per million mapped reads (FPKM) using Cuffdiff (version 2.2.1). The FPKM values were normalized, and statistical analyses were performed on the fold change values using a Student’s *t*-test at *P* < 0.05. The clustered DEGs based on their log_2_ FPKM values were plotted using the R package pheatmap (version 1.0.12). The DEGs were identified from three replicates at each ripening stage.

### Quantitative polymerase chain reaction (qPCR) analysis

Primer sets were designed using the NCBI PrimerBLAST. Sequences of the forward and reverse primers used for the qPCR are listed in S1 Table. The relative expression levels of the transcripts were determined using a Rotor-Gene Q (Qiagen, Valencia, CA, USA) and a Rotor-Gene SYBR Green PCR kit (Qiagen). The results were standardized to the expression level of the gene encoding glyceraldehyde 3-phosphate dehydrogenase, as described by Zifkin et al. [16]. The relative expression levels were plotted using the Prism program (version 8.0.2; GraphPad Software Inc., San Diego, CA, USA).

### Statistical analysis

Statistically significant differences among means were determined by Student’s *t*-test at *P* < 0.05 using the R 3.2.2 software package (http://www.r-project.org).

## Results and discussion

### Fruit skin coloration and anthocyanin accumulation during ripening

The skin color of the ‘Bluecrop’ highbush blueberry fruit changed during ripening (Fig 1). With the calyx turning green to purple, the exocarp was mostly tinted red at reddish purple stage and then shifted bluer at dark purple stage (Fig 1). The reddish purple and dark purple stages indicated fruit at turning point and fully ripe stages, respectively [32]. Our previous study revealed that the skin coloration of ‘Bluecrop’ highbush blueberry fruit during ripening correlated with the accumulation of anthocyanins, especially of delphinidin and delphinidin derivatives [18].

### ABA as a positive regulator of anthocyanin accumulation during ripening

**Fig 2:**
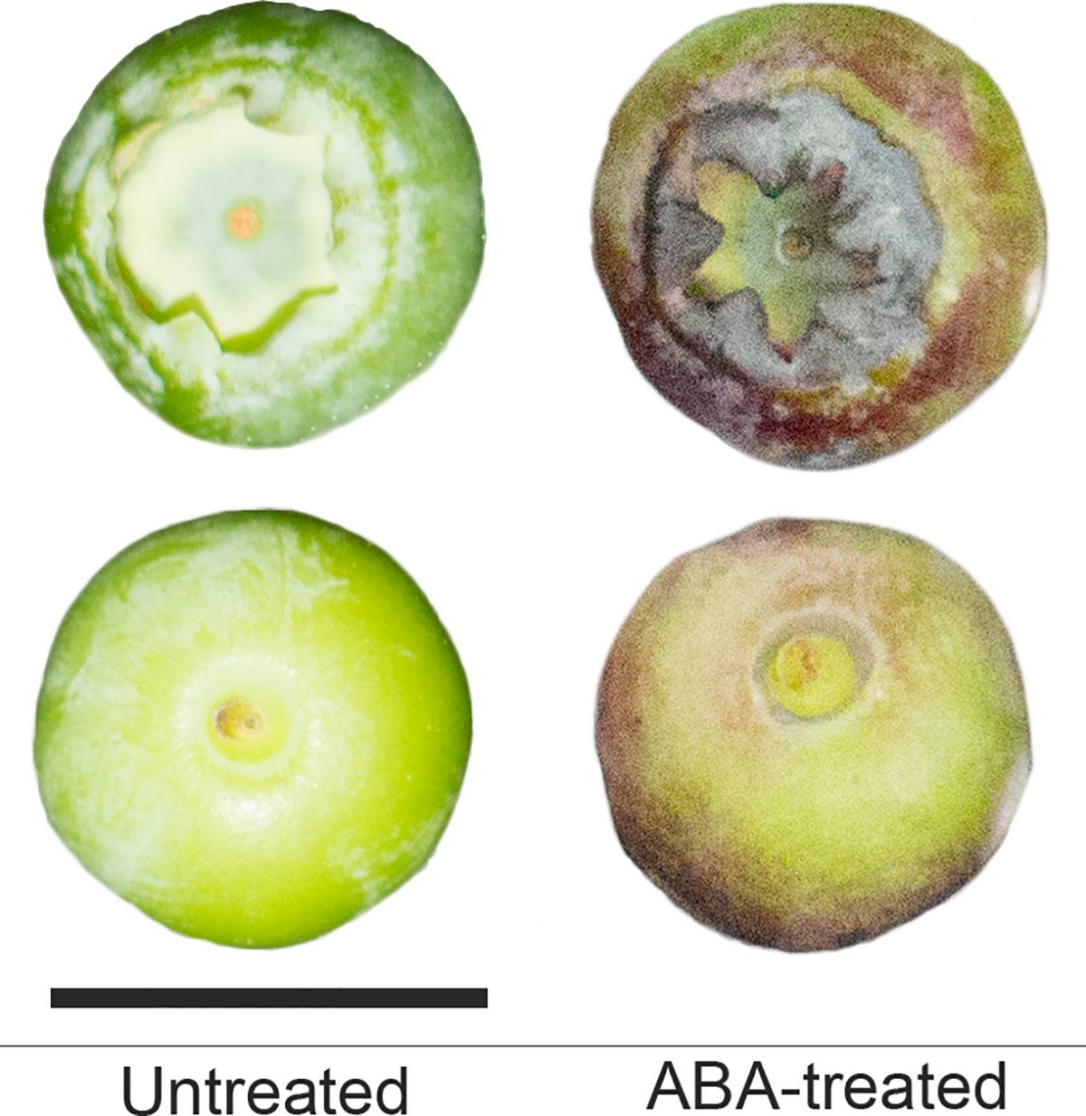
**Calyx end (top) and stem end (bottom) of ‘Bluecrop’ highbush blueberry fruit at 5 days after treatment with or without 1 g L**^**–1**^ **(±) ABA at pale green stage (ca. 30 days after full bloom).** Bar = 1 cm.

In the ABA-treated fruit, the calyx turned dark purple and the exocarp changed to red or purple at 5 DAT, but untreated fruit remained green (Fig 2). Although the L* value of the ABA-treated fruit was not significantly different from that of untreated fruit, the a* and b* values of the ABA-treated fruit were significantly higher and lower, respectively, than those of untreated fruit (Table 1). These results indicated that the ABA-treated fruit were redder and bluer than untreated fruit. Accelerated skin coloration by ABA application has also been reported in other non-climacteric fruits, including strawberry [12, 33], grape [10, 17, 27], and sweet cherry [14].

**Table 1:** Chromaticity values of ‘Bluecrop’ highbush blueberry fruit at 5 days after the treatment with or without 1 g L^–1^ (±) ABA at pale green stage (ca. 30 days after full bloom).

Treatment	L*	a*	b*
Untreated	75.7 ± 5.52^1^ a^2^	–15.1 ± 3.55 b	25.1 ± 1.33 a
ABA-treated	60.7 ± 8.11 a	20.2 ± 6.12 a	15.3 ± 2.11 b

^1^Means with standard errors from three replicates with 30 fruit each.

^2^Means within columns followed by different letters are significantly different according to Student’s *t*-test at *P* < 0.05.

The ABA application also accelerated the accumulation of individual anthocyanins in ‘Bluecrop’ highbush blueberry fruit (Table 2). At 5 DAT, no anthocyanins were detected in untreated fruit, while the ABA-treated fruit accumulated four anthocyanins: cyanidin, malvidin, delphinidin, and petunidin 3-*O*-glucosides (Table 2). However, neither pelargonidin nor peonidin 3-*O*-glucosides were found in the ABA-treated fruit (Table 2). According to our previous study in ‘Bluecrop’ highbush blueberry fruit [18], cyanidins, malvidins, and delphinidins began to accumulate from reddish purple stage and petunidins were accumulated at dark purple stage, but no pelargonidins were accumulated throughout the entire ripening stages. No pelargonidin accumulation was also observed in ‘Jersey’ highbush blueberry fruit regardless of ABA application [8]. The absence of pelargonidin is a common characteristic of the fruits of the Ericaceae [34, 35]. The ABA application promoted the accumulation of anthocyanins, especially of delphinidin derivatives, with a temporary increase in ABA content and thus accelerated fruit skin coloration [8].

**Table 2:** Individual anthocyanin contents in ‘Bluecrop’ highbush blueberry fruit at 5 days after the treatment with or without 1 g L^–1^ (±) ABA at pale green stage (ca. 30 days after full bloom).

Treatment	Cya-glu	Del-glu	Mal-glu	Pel-glu	Peo-glu	Pet-glu
	(μg g^–1^ FW)					
Untreated	nd^1^	nd	nd	nd	nd	nd
ABA-treated	80 ± 14.1^2^	11 ± 3.4	35 ± 4.4	nd	nd	2 ± 0.9

Cya-glu, cyanidin 3-*O*-glucoside; Del-glu, delphinidin 3-*O*-glucoside; Mal-glu, malvidin 3-*O*-glucoside; Pel-glu, pelargonidin 3-*O*-glucoside; Peo-glu, peonidin 3-*O*-glucoside; Pet-glu, petunidin 3-*O*-glucoside.

^1^Not detected.

^2^Means with standard errors from three replicates with 30 fruit each.

### Transcriptome and GO analyses

As the results of RNA-Seq in ‘Bluecrop’ highbush blueberry fruit during ripening, the trimmed reads ranged from 25,552,078 to 28,546,644 with Q30 of 94.9 to 98.2% and GC content of 48.0 to 48.9% (Table 3). The total bases of average 2.58 to 2.88 × 10^9^ were obtained (Table 3).

**Table 3:** RNA-Seq results of ‘Bluecrop’ highbush blueberry fruit at three ripening stages based on skin coloration: pale green at ca. 30 days after full bloom (DAFB), reddish purple at ca. 40 DAFB, and dark purple at ca. 50 DAFB.

Ripening stage	Total read	Total base	Q30 (%)	GC content (%)
Pale green	28,546,644	2,883,211,044	95.3	48.9
Reddish purple	25,552,078	2,580,878,759	94.9	48.6
Dark purple	27,439,060	2,771,345,060	98.2	48.0

Average read length is 101 bp, and each value is the mean from three replicates with 30 fruit each.

**Fig 3:**
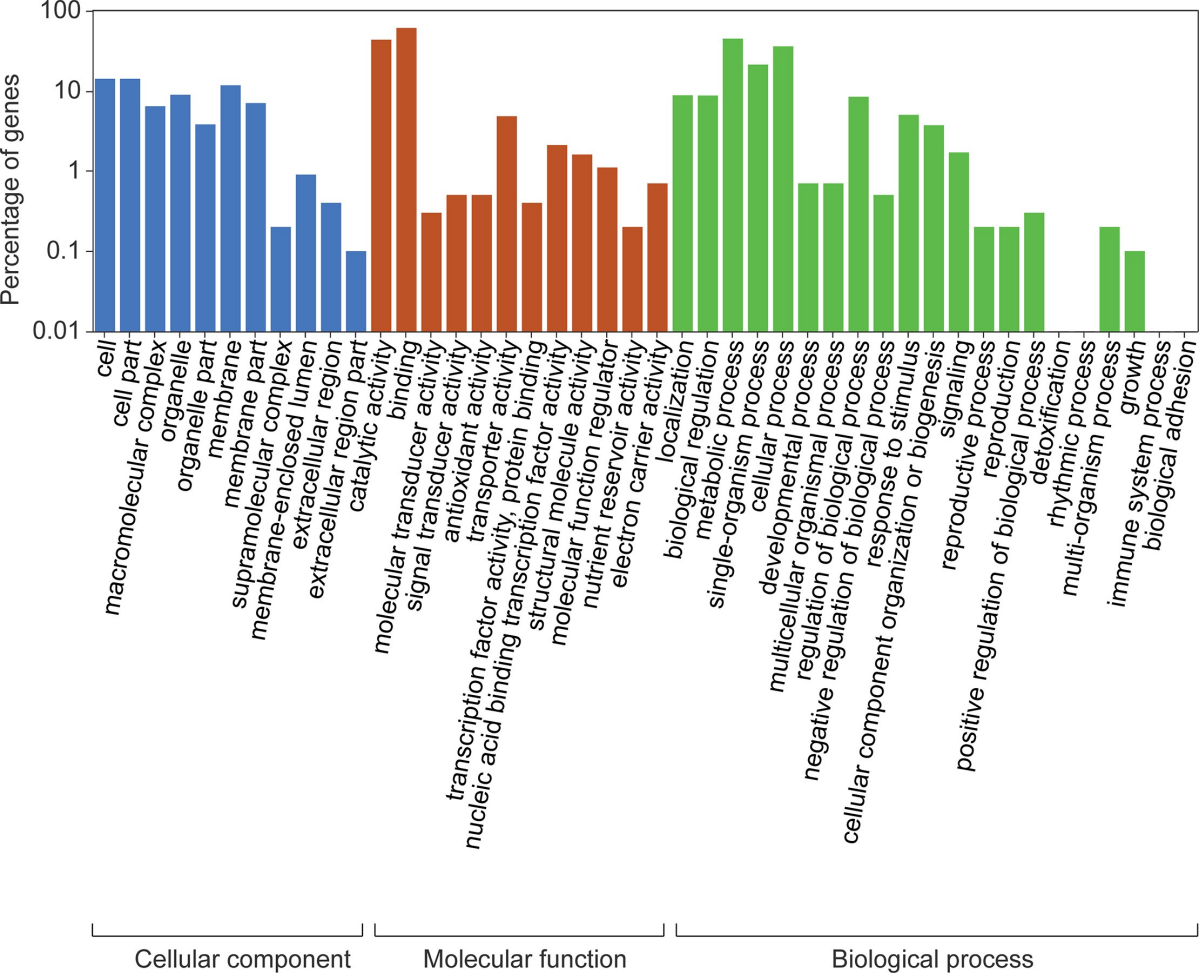
Functional annotation of the transcripts of ‘Bluecrop’ highbush blueberry fruit based on gene ontology categorization into the three main categories: Cellular component, molecular function, and biological process. The y-axis indicates the percentage of genes, expressed as a log_10_ scale.

Of the 25,766 assembled transcripts, 10,998 transcripts were assigned and classified into 44 groups within the three GO categories: cellular component, molecular function, and biological process (Fig 3). The majority of the GO terms (51.7%) were assigned to molecular function, while 34.3 and 14.0% were assigned to biological process and cellular component, respectively (Fig 3). Transcripts associated with binding and catalytic activity were typical in molecular function, while those associated with cell and cell part were highly represented in cellular component. For biological process, metabolic process and cellular process were the most highly represented groups. These two dominant groups in each GO category for ‘Bluecrop’ highbush blueberry fruit (Fig 3) have also been observed in the same cultivar [36] and in other cultivars of ‘Northland’ [23] and ‘O’Neal’ [24].

### Functional annotation of the transcripts involved in ABA biosynthesis and their DEG analysis

**Fig 4:**
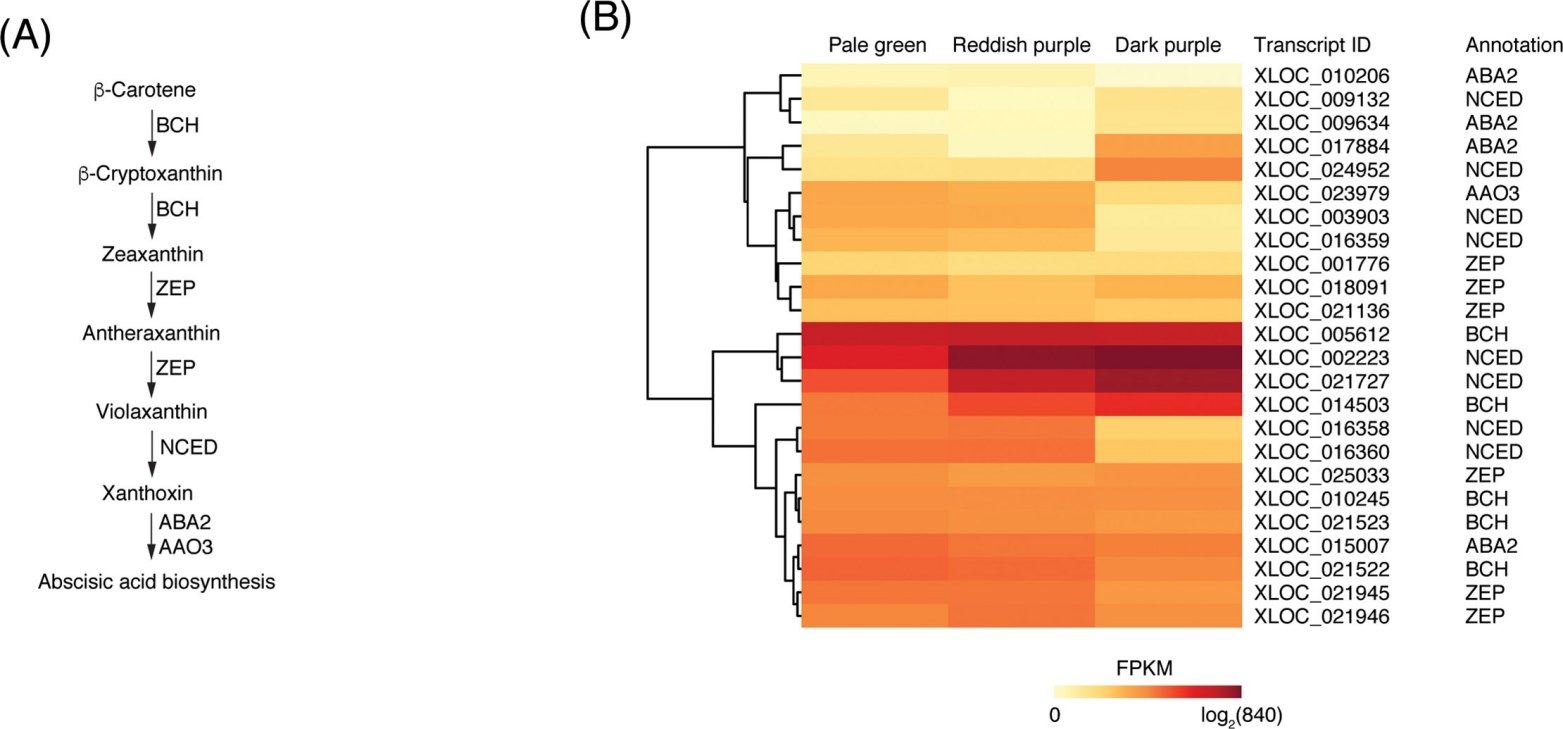
Transcripts involved in abscisic acid (ABA) biosynthesis in ‘Bluecrop’ highbush blueberry fruit during ripening. (A) Schematic view of the ABA biosynthesis pathway (modified from Nambara and Marion-Poll [37]). (B) Heatmap of the log_2_ FPKM expression of candidate transcripts involved in ABA biosynthesis among the three ripening stages based on fruit skin coloration: pale green at ca. 30 days after full bloom (DAFB), reddish purple at ca. 40 DAFB, and dark purple at ca. 50 DAFB. The differentially expressed genes were clustered based on their FPKM values. BCH, ß-carotene 3-hydoroxylase; ZEP, zeaxanthin epoxidase; NCED, nine-*cis*-epoxycarotenoid dioxygenase; ABA2, xanthoxin dehydrogenase; AAO3, abscisic-aldehyde oxidase.

An intermediate of terpenoid pathway, ß-carotene, is sequentially converted to form ABA by the actions of enzymes, such as BCH, zeaxanthin epoxidase (ZEP), NCED, xanthoxin dehydrogenase (ABA2), and abscisic-aldehyde oxidase (AAO3) (Fig 4A) [37].

In the present study, twenty four transcripts were annotated to encode five enzymes involved in ABA biosynthesis: five *BCH*s, six *ZEP*s, eight *NCED*s, four *ABA2*s, and one *AAO3* (Fig 4). The expressions of one *BCH* (XLOC_014503), six *NCED*s (XLOC_002223, XLOC_003903, XLOC_016360, XLOC_016358, XLOC_021727, and XLOC_024952), and one *AAO3* (XLOC_023979) were significantly up- or down-regulated during ripening, while the others were not significantly regulated (S2 Table).

The *BCH* was significantly up-regulated from pale green to reddish purple stages (S2 Table), as observed in kiwifruit [15]. However, ABA accumulation was significantly reduced in two allelic *dsm2* mutants of rice, which lacked a functional BCH protein [38]. NCEDs and their isoforms have been identified in many plant species, including bilberry [17, 39, 40], tomato [41, 42], and grape [43, 44, 45]. These enzymes were differentially expressed depending on tissues, developmental stages, and environmental conditions [46]. *NCED* expression was reported to temporarily increase with the increased ABA contents during ripening of grape [9] and ‘Rubel’ highbush blueberry fruits [16]. Of the six *NCED*s, two *NCED*s (XLOC_002223 and XLOC_021727) were also significantly up-regulated from pale green to reddish purple stages (S2 Table). From reddish purple to dark purple stages, one *NCED* (XLOC_024952) was up-regulated, while the remaining three *NCED*s were down-regulated (S2 Table). The *AAO3* was significantly down-regulated from reddish purple to dark purple stages, but its FPKM values remained low throughout the entire ripening stages (Fig 4B).

### Functional annotation of the transcripts involved in ABA signal transduction and their DEG analysis

**Fig 5:**
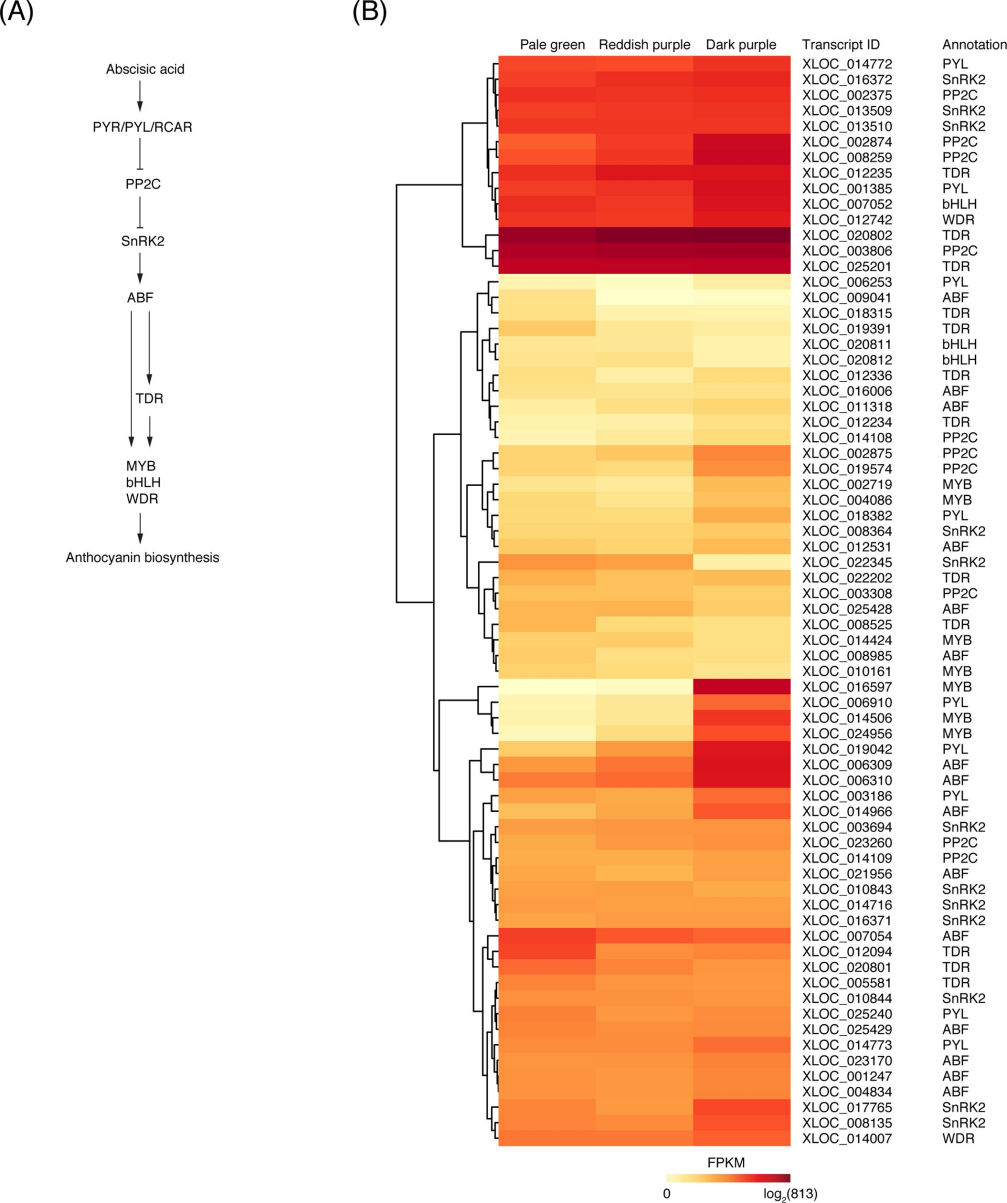
Transcripts involved in abscisic acid (ABA) signal transduction in ‘Bluecrop’ highbush blueberry fruit during ripening. (A) Schematic view of the ABA signal transduction pathway (modified from Li et al. [48]). (B) Heatmap of the log_2_ FPKM of candidate transcripts involved in ABA signal transduction among the three ripening stages based on fruit skin coloration: pale green at ca. 30 days after full bloom (DAFB), reddish purple at ca. 40 DAFB, and dark purple at ca. 50 DAFB. The differentially expressed genes were clustered based on their FPKM values. PYR/PYL/RCAR, pyrabactin resistance/pyrabactin resistance-like/regulatory components of ABA receptors; PP2C, protein phosphatases type 2C; SnRK2, sucrose non-fermenting-1-related protein kinase 2; ABF, ABA-responsive element binding factor; TDR, SQUAMOSA-class MADS box transcription factor; MYB, R2R3MYB transcription factor; bHLH, basic helix-loop-helix transcription factor; WDR, β-transduction repeat transcription factor.

In ABA signal transduction (Fig 5A), ABA activates PYR/PYL/RCAR [43], and then the ABA-activated PYR/PYL/RCAR inhibits a protein phosphatase 2C (PP2C) [47], leading to the activation of sucrose non-fermenting-1-related protein kinase 2 (SnRK2). The SnRK2 regulates the ABA-responsive element binding factor (ABF) [43, 48].

In the present study, 46 transcripts were annotated to encode four signal transduction regulators (nine *PYL*s, ten *PP2C*s, 12 *SnRK2*s, and 15 *ABF*s) (Fig 5). Of the signal transduction regulators, one *PYL* (XLOC_025240) was significantly up-regulated throughout the entire ripening stages, while 14 transcripts (five *PYL*s [XLOC_001385, XLOC_006253, XLOC_014772, XLOC_ 018382, and XLOC_019042], four *PP2C*s [XLOC_002375, XLOC_008259, XLOC_014109, and XLOC_0195742], two *SnRK2*s [XLOC_003694 and XLOC_008135], and three *ABF*s [XLOC_016006, XLOC_021956, and XLOC_023170]) were significantly up-regulated from reddish purple to dark purple stages (S2 Table). However, one *SnRK2* (XLOC_022345) and one *ABF* (XLOC_009041) were down-regulated throughout the entire ripening stages, but the others were not significantly regulated (S2 Table).

In *Arabidopsis*, ABA application induced the transcriptional expression of the PYR/PYL/RCAR family [49]. In strawberry fruit, similarly, ABA application enhanced the up-regulation of a *PYR* among the family during ripening, while no coloration occurred in the *PYR*-silenced fruit regardless of ABA application [5]. However, up-regulation of the four *PP2C*s and down-regulation of the one *SnRK2* and one *ABF* in the present study (S2 Table) were contradictory to the results in *Arabidopsis* [49]. The question of if there is ABA signal transduction process specific for highbush blueberry fruit remains to be answered unequivocally.

ABF in ABA signal transduction (Fig 5A) regulates various transcription factors, such as SQUAMOSA-class MADS box (TDR), R2R3MYB (MYB), basic helix-loop-helix (bHLH), and ß-transduction repeat (WDR), which are associated with the gene expressions in anthocyanin biosynthesis [17, 39, 50].

In the present study, 21 transcripts were annotated to encode four ABA-responsive transcription factors (ten *TDR*s, seven *MYB*s, two *bHLH*s, and two *WDR*s) (Fig 5). Of the ABA-responsive transcription factors, *TDR* (XLOC_020802) was most highly expressed throughout the entire ripening stages (Fig 5B). However, two *TDR*s (XLOC_008525 and XLOC_012094) were significantly down-regulated from pale green to reddish purple stages and their expressions remained low thereafter (S2 Table). Four *MYB*s (XLOC_002719, XLOC_004086, XLOC_014506, and XLOC_024956) and one *bHLH* (XLOC_007502) were significantly up-regulated from reddish purple to dark purple stages, but the others were not significantly regulated throughout the entire ripening stages (S2 Table).

In bilberry fruit, *TDR* and *MYB* were found to be sequentially up-regulated during ripening and the up-regulations were enhanced by ABA application, increasing the anthocyanin accumulation [39]. ABA application also enhanced the expressions of *bHLH* as well as *MYB* in grape fruit [51]. Silencing *TDR* and *MYB* led to a decrease in anthocyanin accumulation and its associated gene expressions in sweet cherry [14] and bilberry fruits [39].

### Functional annotation of the transcripts involved in anthocyanin biosynthesis and their DEG analysis

**Fig 6:**
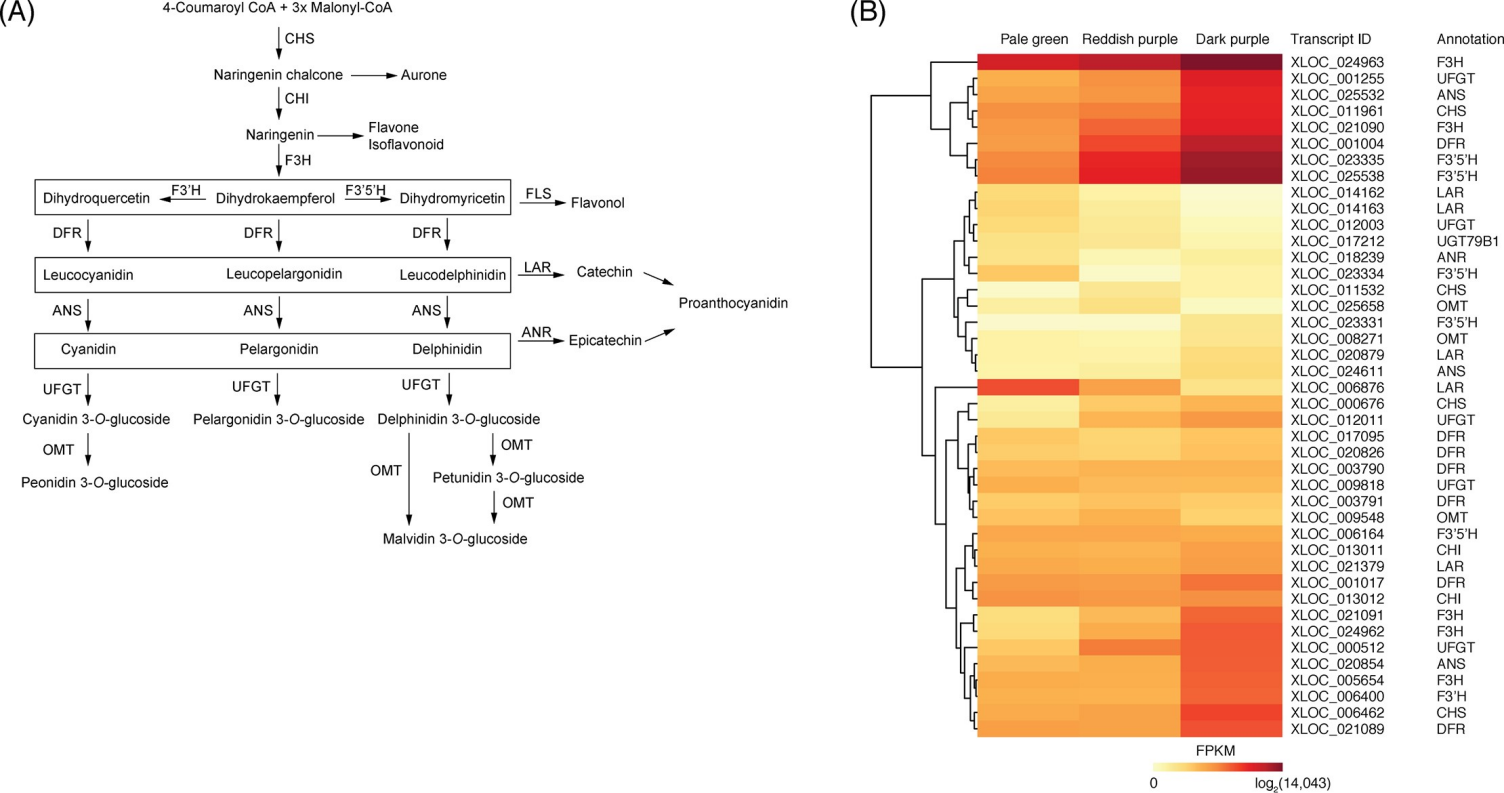
Transcripts involved in anthocyanin biosynthesis in ‘Bluecrop’ highbush blueberry fruit during ripening. (A) Schematic view of the anthocyanin biosynthesis pathway (modified from Zifkin et al. [16]). (B) Heatmap of the log_2_ FPKM expression of candidate transcripts involved in anthocyanin biosynthesis among the three ripening stages based on fruit skin coloration: pale green at ca. 30 days after full bloom (DAFB), reddish purple at ca. 40 DAFB, and dark purple at ca. 50 DAFB. The differentially expressed genes were clustered based on their FPKM values. CHS, chalcone synthase; CHI, chalcone isomerase; F3H, flavanone 3-hydroxylase; F3′H, flavonoid 3′-hydroxylase; F3′5′H, flavonoid 3′,5′-hydroxylase; DFR, dihydroflavonol 4-reductase; ANS, anthocyanidin synthase; UFGT, anthocyanin 3-*O*-glucosyltransferase; OMT, *O*-methyltransferase; FLS, flavonol synthase; LAR, leucoanthocyanidin reductase; ANR, anthocyanidin reductase.

Anthocyanins are biosynthesized by the sequential actions of enzymes, such as chalcone synthase (CHS), chalcone isomerase (CHI), flavanone 3-hydroxylase (F3H), flavonoid 3′-hydroxylase (F3′H), flavonoid 3′,5′-hydroxylase (F3′5′H), dihydroflavonol 4-reductase (DFR), anthocyanidin synthase (ANS), anthocyanin 3-*O*-glucosyltransferase (UFGT), and *O*-methyltransferase (OMT) (Fig 6A) [16, 52]. These enzymes and their related genes in anthocyanin biosynthesis have been discovered in several plant species, including *Arabidopsis*, maize, grape, and petunia [53], and the anthocyanin biosynthesis pathway was reported to be highly conserved in angiosperms [54].

In the present study, 42 transcripts were annotated to encode 12 enzymes involved in anthocyanin biosynthesis: four *CHS*s, two *CHI*s, six *F3H*s, one *F3′H*, five *F3′5′H*s, six *DFR*s, three *ANS*s, five *UFGT*s, three *OMT*s, five *leucoanthocyanidin reductases* (*LAR*s), one *anthocyanidin reductase* (*ANR*), and one *anthocyanidin 3-O-glucoside 2′′′*-*O-xylosyltransferase* (*UGT79B1*) (Fig 6).

Of the 25 significantly regulated transcripts, ten transcripts (one *CHS* [XLOC_000676], three *F3H*s [XLOC_021090, XLOC_021091, and XLOC_024962], two *F3′5′H*s [XLOC_023335 and XLOC_025538], one *DFR* [XLOC_017095], and three *UFGT*s [XLOC_000512, XLOC_001255, and XLOC_012011]) were up-regulated from pale green to dark purple stages, while another ten transcripts (two *CHS*s [XLOC_006462 and XLOC_011961], one *CHI* [XLOC_013011], three *F3H*s [XLOC_005654, XLOC_021089, and XLOC_024963], one *F3′H* [XLOC_006400], one *DFR* [XLOC_001004], and two *ANS*s [XLOC_024611 and XLOC_025532]) were up-regulated from reddish purple to dark purple stages (S2 Table). In addition, one *OMT* (XLOC_009548), three *LAR*s (XLOC_006876, XLOC_014163, and XLOC_020879), and one *ANR* (XLOC_018239) were down-regulated throughout the entire ripening stages (S2 Table).

The transcripts encoding CHS, CHI, and F3H, which produce common anthocyanin precursors (Fig 6A), were all up-regulated throughout the entire ripening stages (S2 Table), as observed in peach [22], sweet cherry [14], bilberry [39], and ‘Rubel’ highbush blueberry fruits [16].

The cytochrome P450-dependent monooxygenases F3′H and F3′5′H determine the types of anthocyanins [55, 56]. The F3′H and F3′5′H hydroxylate the B-ring of anthocyanidin skeletons at the 3′-, and 3′- and 5′-positions, respectively. The F3′H and F3′5′H are associated with the accumulations of cyanidin and delphinidin derivatives, respectively (Fig 6A). Cyanidin and its methoxylated derivative peonidin confer red colors, while delphinidin and its methoxylated derivatives petunidin and malvidin are the main contributors of purple and blue colors [57]. The anthocyanins except pelargonidins were abundantly found in ripe fruit of highbush blueberry [18, 58]. In the present study, the two *F3′5′H*s (XLOC_023335 and XLOC_025538) were more highly expressed than *F3′H* (XLOC_006400) (Fig 6B). The higher expression of *F3′5′H* than *F3′H* was consistent with the higher accumulation of the delphinidin derivatives than the cyanidin derivatives in several highbush blueberry cultivars [18, 58]. Since an increase in the number of hydroxyl groups increases the blueness of the anthocyanins [55, 57], the expressions of the *F3′H* and *F3′5′H* (Fig 6B) might lead to purple or blue skin coloration in ‘Bluecrop’ highbush blueberry fruit during ripening (Fig 1).

Following the hydroxylation, individual anthocyanins are biosynthesized at different levels depending on the reactions of DFR and ANS with their respective substrates [52, 53]. In strawberry fruit, one DFR isoform specifically reacted with dihydrokaempferol to biosynthesize pelargonidin derivatives, the most abundant anthocyanins in these fruit [59].

Glucosylation, galactosylation, and arabinosylation were the major glycosylation processes, which were catalyzed by the actions of various sugar transferases, in highbush blueberry fruit [8, 18]. In our previous study, we determined 22 anthocyanins resulted from these three glycosylation processes in ‘Bluecrop’ highbush blueberry fruit [18]. In the present study, however, three out of five transcripts encoding UFGTs for glucosylation were up-regulated throughout the entire ripening stages (S2 Table), but the transcripts associated with galactosylation and arabinosylation were not found (Fig 6B). Similarly to our report, no transcripts associated with galactosylation or arabinosylation have been found in the other transcriptome analyses of highbush blueberry fruit [23–27].

Leucoanthocyanidins and anthocyanidins can be diverted to flavan 3-ols by the actions of LAR and ANR, respectively [16]. Since LAR and ANR compete with ANS and UFGT for their respective common substrates, the up-regulations of *ANS*s and *UFGT*s and the down-regulations of *LARs* and *ANR* (S2 Table) might also contribute to anthocyanin accumulation in highbush blueberry fruits during ripening. Following the glycosylation, OMT methoxylates the B-ring of anthocyanidin skeletons at the 3′-, and 3′- and 5′-positions [51, 60]. The transcript encoding OMT was found to be down-regulated in ‘Bluecrop’ highbush blueberry fruit throughout the entire ripening stages (S2 Table) as observed in grape fruit, despite of the accumulation of methoxylated anthocyanins [57].

### Transcriptional expression in ABA-treated fruit

**Fig 7:**
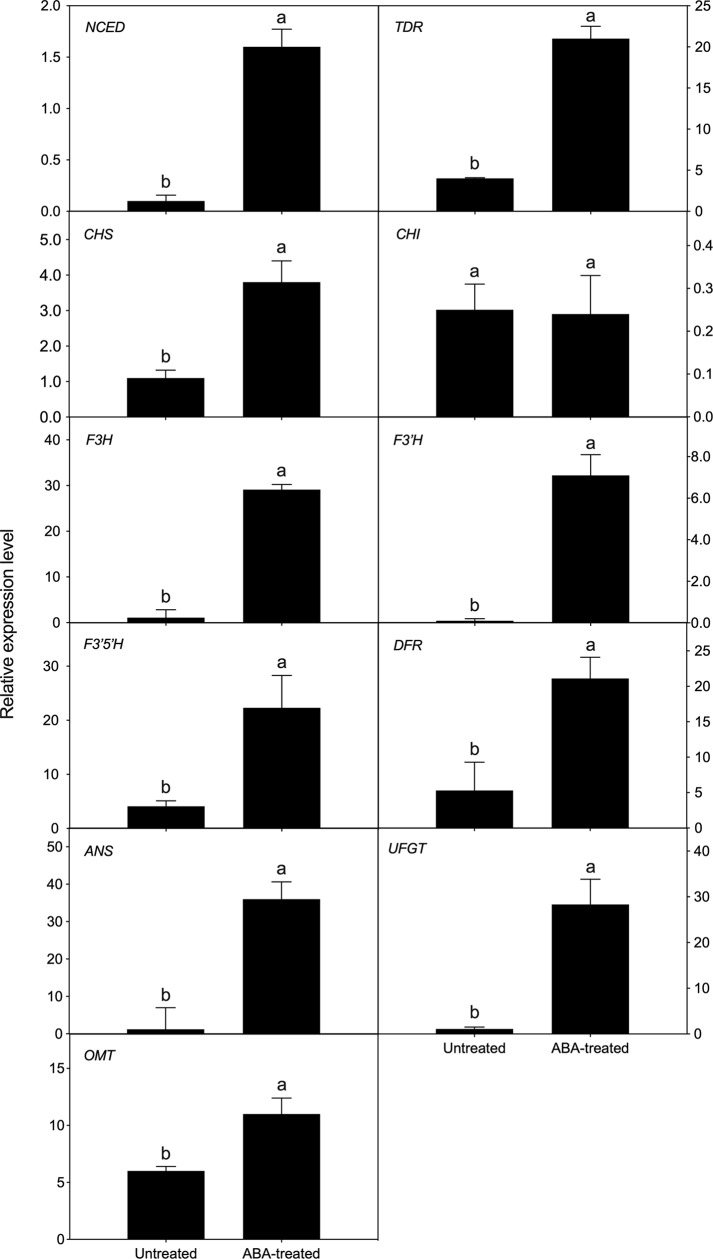
Relative gene expression in ‘Bluecrop’ highbush blueberry fruit at 5 days after treatment with or without 1 g L^–1^ (±) ABA at pale green stage (ca. 30 days after full bloom). Means with bars followed by different letters are significantly different according to Student’s *t*-test at *P* < 0.05. Vertical bars represented standard errors of means (n = 3). *NCED*, nine-*cis*-epoxycarotenoid dioxygenase (XLOC_002223); *TDR*, SQUAMOSA-class MADS box transcription factor (XLOC_020802); *CHS*, chalcone synthase (XLOC_011961); *CHI*, chalcone isomerase (XLOC_013012); *F3H*, flavanone 3-hydroxylase (XLOC_024963); *F3′H*, flavonoid 3′-hydroxylase (XLOC_006400); *F3′5′H*, flavonoid 3′,5′-hydroxylase (XLOC_025538); *DFR*, dihydroflavonol 4-reductase (XLOC_001004); *ANS*, anthocyanidin synthase (XLOC_025532); *UFGT*, anthocyanin 3-*O*-glucosyltransferase (XLOC_001255); *OMT*, *O*-methyltransferase (XLOC_008271).

The effects of ABA treatment on the transcript expression were confirmed by qPCR analysis against the annotated transcripts, which were found to be highly up-regulated during ripening. All transcripts examined except for *CHI* (XLOC_013012) were more highly expressed in the ABA-treated fruit at 5 DAT than in untreated fruit (Fig 7). These included *NCED* (XLOC_002223), *TDR* (XLOC_020802), *CHS* (XLOC_011961), *F3H* (XLOC_024963), *F3′H* (XLOC_006400), *F3′5′H* (XLOC_025538), *DFR* (XLOC_001004), *ANS* (XLOC_025532), *UFGT* (XLOC_001255), and *OMT* (XLOC_008271).

The ABA-mediated up-regulation of *NCED* in ‘Bluecrop’ highbush blueberry fruit (Fig 7) was also previously observed in other non-climacteric fruits, including grape [43], strawberry [12], and bilberry [17], implying that the endogenous ABA contents increased during ripening. Exogenous ABA as well as the increased endogenous ABA might accelerate the ripening process, including fruit skin coloration and cell softening.

The up-regulations of *TDR* and eight genes (*CHS*, *F3H*, *F3′H*, *F3′5′H*, *DFR*, *ANS*, *UFGT*, and *OMT*) (Fig 7) might contribute to the anthocyanin accumulation (Table 2) and the associated fruit skin coloration (Fig 2 and Table 1). In ABA-treated grape [61], strawberry [12], and blueberry fruits [8], however, the levels of anthocyanin accumulation and its associated gene expression were dependent on the concentration, timing, and duration of the ABA application. Despite these differences, the transcription factors and genes were similarly expressed in those fruits during ripening [8, 12, 61, 62]. More detailed information on ABA effects under various environmental and experimental conditions is needed to understand the ripening process of naturally grown fruits and to explore the possible implementation of ABA application in agricultural fields.

## Supporting information

S1 Table Sequences of forward and reverse primers used for quantitative polymerase chain reaction. (XLSX)

S2 Table Fold changes in the transcriptional expression involved in abscisic acid biosynthesis and signal transduction, and anthocyanin biosynthesis in ‘Bluecrop’ highbush blueberry fruit during ripening. (XLSX)

## References

[1] Giovannoni J. Molecular biology of fruit maturation and ripening. Annu Rev Plant Physiol. 2001; 52:725–49. 10.1146/annurev.arplant.52.1.725 11337414

[2] Kondo S, Meemak S, Ban Y, Moriguchi T, Harada T. Effects of auxin and jasmonates on 1-aminocyclopropane-1-carboxylate (ACC) synthase and ACC oxidase gene expression during ripening of apple fruit. Postharvest Biol Technol. 2009; 51(2):281–4. 10.1016/j.postharvbio.2008.07.012

[3] Xu F, Yuan S, Zhang DW, Lv X, Lin HH. The role of alternative oxidase in tomato fruit ripening and its regulatory interaction with ethylene. J Exp Bot. 2012; 63(15):5705–16. 10.1093/jxb/ers226 22915749 PMC3444281

[4] Frenkel C. Involvement of peroxidase and indole-3-acetic acid oxidase isoenzymes from pear, tomato, and blueberry fruit in ripening. Plant Physiol. 1972; 49(5):757–63. 10.1104/pp.49.5.757 16658043 PMC366047

[5] Chai YM, Jia HF, Li CL, Dong QH, Shen YY. FaPYR1 is involved in strawberry fruit ripening. J Exp Bot. 2011; 62(14):5079–89. 10.1093/jxb/err207 21778181

[6] Symons GM, Chua YJ, Ross JJ, Quittenden LJ, Davies NW, Reid JB. Hormonal changes during non-climacteric ripening in strawberry. J Exp Bot. 2012; 63(13):4741–50. 10.1093/jxb/ers147 22791823 PMC3428006

[7] Chen J, Mao L, Lu W, Ying T, Luo Z. Transcriptome profiling of postharvest strawberry fruit in response to exogenous auxin and abscisic acid. Planta. 2016; 243(1):183–97. 10.1007/s00425-015-2402-5 26373937

[8] Oh HD, Yu DJ, Chung SW, Chea S, Lee HJ. Abscisic acid stimulates anthocyanin accumulation in ‘Jersey’ highbush blueberry fruits during ripening. Food Chem. 2018; 244(1):403–7. 10.1016/j.foodchem.2017.10.051 29120800

[9] Wheeler S, Loveys B, Ford C, Davies C. The relationship between the expression of abscisic acid biosynthesis genes, accumulation of abscisic acid, and the promotion of *Vitis vinifera* L. berry ripening by abscisic acid. Aust J Grape Wine Res. 2009; 15(3):195–204. 10.1111/j.1755-0238.2008.00045.x

[10] Koyama K, Sadamatsu K, Goto-Yamamoto N. Abscisic acid stimulated ripening and gene expression in berry skins of the Cabernet Sauvignon grape. Funct Integr Genom. 2010; 10(3):367–81. 10.1007/s10142-009-0145-8 19841954

[11] Villalobos-González L, Peña-Neira A, Ibáñez F, Pastenes C. Long-term effects of abscisic acid (ABA) on the grape berry phenylpropanoid pathway: gene expression and metabolite content. Plant Physiol Biochem. 2016; 105:213–23. 10.1016/j.plaphy.2016.04.012 27116369

[12] Jia HF, Chai YM, Li CL, Lu D, Luo JJ, Qin L, et al Abscisic acid plays an important role in the regulation of strawberry fruit ripening. Plant Physiol. 2011; 157(1):188–99. 10.1104/pp.111.177311 21734113 PMC3165869

[13] Luo H, Dai S, Ren J, Zhang C, Ding Y, Li Z, et al The role of ABA in the maturation and postharvest life of a non-climacteric sweet cherry fruit. J Plant Growth Regul. 2014; 33(2):373–83. 10.1007/s00344-013-9388-7

[14] Shen X, Zhao K, Liu L, Zhang K, Yuan H, Liao X, et al A role for PacMYBA in ABA-regulated anthocyanin biosynthesis in red-colored sweet cherry cv. Hong Deng (*Prunus avium* L.). Plant Cell Physiol. 2014; 55(5):862–80. 10.1093/pcp/pcu013 24443499

[15] Ampomah-Dwamena C, McGhie T, Wibisono R, Montefiori M, Hellens RP, Allen AC. The kiwifruit lycopene ß-cyclase plays a significant role in carotenoid accumulation in fruit. J Exp Bot. 2009; 60(13):3765–79. 10.1093/jxb/erp218 19574250 PMC2736891

[16] Zifkin M, Jin A, Ozga JA, Zaharia LI, Schernthaner JP, Gesell A, et al Gene expression and metabolite profiling of developing highbush blueberry fruit indicates transcriptional regulation of flavonoid metabolism and activation of abscisic acid metabolism. Plant Physiol. 2012; 158(1):200–24. 10.1104/pp.111.180950 22086422 PMC3252089

[17] Karppinen K, Tegelberg P, Haggman H, Jaakola L. Abscisic acid regulates anthocyanin biosynthesis and gene expression associated with cell wall modification in ripening bilberry (*Vaccinium myrtillus* L.) fruits. Front Plant Sci. 2018; 9:1259 10.3389/fpls.2018.01259 30210522 PMC6124387

[18] Chung SW, Yu DJ, Lee HJ. Changes in anthocyanidin and anthocyanin pigments in highbush blueberry (*Vaccinium corymbosum* cv. Bluecrop) fruits during ripening. Hortic Environ Biotechnol. 2016; 57(5):424–30. 10.1007/s13580-016-0107-8

[19] Wu X, Prior RL. Systematic identification and characterization of anthocyanins by HPLC-ESI-MS/MS in common foods in the United States: fruits and berries. J Agric Food Chem. 2005; 53(7):5789–99. 10.1021/jf048068b 15796599

[20] Wang Z, Gerstein M, Snyder M. RNA-Seq: a revolutionary tool for transcriptomics. Nat Rev Genet. 2009; 10(1):57–63. 10.1038/nrg2484 19015660 PMC2949280

[21] Ozsolak F, Milos PMM. RNA sequencing: advances, challenges, and opportunities. Nat Rev Genet. 2011; 12(2):87–98. 10.1038/nrg2934 21191423 PMC3031867

[22] Cao K, Ding T, Mao D, Zhu G, Fang W, Chen C, et al Transcriptome analysis reveals novel genes involved in anthocyanin biosynthesis in the flesh of peach. Plant Physiol Biochem. 2017; 123:94–102. 10.1016/j.plaphy.2017.12.005 29227951

[23] Li XY, Sun HY, Pei JB, Dong YY, Wang FW, Chen H, et al De novo sequencing and comparative analysis of the blueberry transcriptome to discover putative genes related to antioxidants. Gene. 2012; 511(1):54–61. 10.1016/j.gene.2012.09.021 22995346

[24] Gupta V, Estrada AD, Blakley I, Reid R, Patel K, Meyer MD, et al RNA-Seq analysis and annotation of a draft blueberry genome assembly identifies candidate genes involved in fruit ripening, biosynthesis of bioactive compounds, and stage-specific alternative splicing. Gigascience. 2015; 13(4):5 10.1186/s13742-015-0046-9 25830017 PMC4379747

[25] Li LL, Zhang HH, Liu ZS, Cui XY, Zhang T, Li YF, et al Comparative transcriptome sequencing and de novo analysis of *Vaccinium corymbosum* during fruit and color development. BMC Plant Biol. 2016; 16(1):223 10.1186/s12870-016-0866-5 27729032 PMC5059916

[26] Lin Y, Wang Y, Li B, Tan H, Li D, Li L, et al Comparative transcriptome analysis of genes involved in anthocyanin synthesis in blueberry. Plant Physiol Biochem. 2018; 127:561–72. 10.1016/j.plaphy.2018.04.034 29727860

[27] Jeong ST, Goto-Yamamoto N, Kobayashi S, Esaka M. Effects of plant hormones and shading on the accumulation of anthocyanins and the expression of anthocyanin biosynthetic genes in grape berry skins. Plant Sci. 2004; 167(2):247–52. 10.1016/j.plantsci.2004.03.021 16556978

[28] Zaharia LI, Walker-Simmon MK, Rodriguez CN, Abrams SR. Chemistry of abscisic acid, abscisic acid catabolites, and analogs. J Plant Growth Regul. 2005; 24(4):274–84. 10.1007/s00344-005-0066-2

[29] McGuire RG. Reporting of objective color measurements. HortScience. 1992; 27(12):1254–5. 10.21273/hortsci.27.12.1254

[30] Gavrilova V, Kajdzanoska M, Gjamovski V, Stefova M. Separation, characterization, and quantification of phenolic compounds in blueberries and red and black currants by HPLC-DAD-ESI-MS*n*. J Agric Food Chem. 2011; 59(8):4009–18. 10.1021/jf104565y 21401099

[31] Jaakola L, Pirttila AM, Halonen M, Hohtola A. Isolation of high quality RNA from bilberry (*Vaccinium myrtillus* L.) fruit. Mol Biotechnol. 2001; 19(2):201–3. 10.1385/MB:19:2:201 11725489

[32] Chea S, Yu DJ, Park J, Oh HD, Chung SW, Lee HJ. Fruit softening correlates with enzymatic and compositional changes in fruit cell wall during ripening in ‘Bluecrop’ highbush blueberries. Sci Hortic. 2019; 245:163–70. 10.1016/j.scienta.2018.10.019

[33] Li D, Luo Z, Mou W, Wang Y, Ying T, Mao L. ABA and UV-C effects on quality, antioxidant capacity, and anthocyanin contents of strawberry fruit (*Fragaria ananassa* Duch.). Postharvest Biol Technol. 2014; 90:56–62. 10.1016/j.postharvbio.2013.12.006

[34] Ribera AE, Reyes-Diaz M, Alberdi M, Zuniga GE, Mora ML. Antioxidant compounds in skin and pulp of fruits change among genotypes and maturity stages in highbush blueberry (*Vaccinium corymbosum* L.) grown in southern Chile. J Soil Sci Plant Nutr. 2010; 10(4):509–36. 10.4067/S0718-95162010000200010

[35] Jaakola L, Maatta K, Pirttila AM, Torronen R, Karenlampi S, Hohtola A. Expression of genes involved in anthocyanin biosynthesis in relation to anthocyanin, proanthocyanidin, and flavonol levels during bilberry fruit development. Plant Physiol. 2002; 130(2):729–39. 10.1104/pp.006957 12376640 PMC166602

[36] Rowland LJ, Alkharoug N, Darwish O, Ogden EL, Polashcok LO, Bassil NV, et al Generation and analysis of blueberry transcriptome sequences from leaves, developing fruit, and flower buds from cold acclimation through deacclimation. BMC Plant Biol. 2012; 12:46 10.1186/1471-2229-12-46 22471859 PMC3378433

[37] Nambara E, Marion-Poll A. Abscisic acid biosynthesis and catabolism. Annu Rev Plant Biol. 2005:165–85. 10.1146/annurev.arplant.56.032604.144046 15862093

[38] Du H, Wang N, Li X, Xio J, Xiong L. Characterization of the ß-carotene hydroxylase gene *DSM2* conferring drought and oxidative stress resistance by increasing xanthophylls and abscisic acid synthesis in rice. Plant Physiol. 2010; 154(3):1304–18. 10.1104/pp.110.163741 20852032 PMC2971608

[39] Jaakola L, Poole M, Jones MO, Kamaraninen-Karppinen T, Koskimaki JJ, Hohtola A, et al A SQUAMOSA MADS box gene involved in the regulation of anthocyanin accumulation in bilberry fruits. Plant Physiol. 2010; 153(4):1619–29. 10.1104/pp.110.158279 20566708 PMC2923880

[40] Karppinen K, Hirvelä E, Nevala T, Sipari N, Suokas M, Jaakola L. Changes in the abscisic acid levels and related gene expression during fruit development and ripening in bilberry (*Vaccinium myrtillus* L.). Phytochemistry. 2013; 95:127–34. 10.1016/j.phytochem.2013.06.023 23850079

[41] Zhang M, Yuan B, Leng P. The role of ABA in triggering ethylene biosynthesis and ripening of tomato fruit. J Exp Bot. 2009; 60(6):1579–88. 10.1093/jxb/erp026 19246595 PMC2671613

[42] Sun L, Sun Y, Zhang M, Wang L, Ren J, Cui M, et al Suppression of 9-*cis*-epoxycarotenoid dioxygenase, which encodes a key enzyme in abscisic acid biosynthesis, alters fruit texture in transgenic tomato. Plant Physiol. 2012; 158(1):283–98. 10.1104/pp.111.186866 22108525 PMC3252109

[43] Pilati S, Bagagli G, Sonego P, Moretto M, Brazzale D, Catorina G. Abscisic acid is a major regulator of grape berry ripening onset: new insights into ABA signaling network. Font Plant Sci. 2017; 8:1093 10.3389/fpls.2017.01093 28680438 PMC5479058

[44] Lund S, Peng F, Nayar T, Reid K, Schlosser J. Gene expression analyses in individual grape (*Vitis vinifera* L.) berries during ripening initiation reveal that pigmentation intensity is a valid indicator of developmental staging within the cluster. Plant Mol Biol. 2008; 68(3):301–15. 10.1007/s11103-008-9371-z 18642093

[45] Young PR, Lashbrooke JG, Alexandersson E, Jacobson D, Moser C, Velasco R, et al The genes and enzymes of the carotenoid metabolic pathway in *Vitis vinifera* L. BMC Genom. 2012; 13:243 10.1186/1471-2164-13-243 22702718 PMC3484060

[46] Chernys JT, Zeevaart JA. Characterization of the 9-*cis*-epoxycarotenoid dioxygenase gene family and the regulation of abscisic acid biosynthesis in avocado. Plant Physiol. 2000; 124(1):343–53. 10.1104/pp.124.1.343 10982448 PMC59148

[47] Park SY, Fung P, Nishimura N, Jensen DR, Fujii H, Zhao Y, et al Abscisic acid inhibits type 2C protein phosphatases via the PYR/PYL family of START proteins. Science. 2009; 324(5930):1068–71. 10.1126/science.1173041 19407142 PMC2827199

[48] Li C, Jia H, Chai Y, Shen Y. Abscisic acid perception and signaling transduction in strawberry: a model for non-climacteric fruit ripening. Plant Signal Behav. 2011; 6(12):1950–3. 10.4161/psb.6.12.18024 22095148 PMC3337185

[49] Yang W, Zhang W, Wang X. Post-transcriptional control of ABA signaling: the roles of protein phosphorylation and ubiquitination. Plant Biotechnol J. 2017; 15(1):4–14. 10.1111/pbi.12652 27767245 PMC5253474

[50] Hu B, Lai B, Wang D, Li J, Chen L, Qin Y, et al Three LcABFs are involved in the regulation of chlorophyll degradation and anthocyanin biosynthesis during fruit ripening in *Litchi chinensis*. Plant Cell Physiol. 2019; 60(2):448–61. 10.1093/pcp/pcy219 30407601

[51] Rattanakon S, Ghan R, Gambetta GA, Deluc LG, Schlauch KA, Cramer GR. Abscisic acid transcriptomic signaling varies with grapevine organ. BMC Plant Biol. 2016; 16:72 10.1186/s12870-016-0763-y 27001301 PMC4802729

[52] Lu Y, Rausher MD. Evolutionary rate variation in anthocyanin pathway genes. Mol Biol Evol. 2003; 20(11):1844–53. 10.1093/molbev/msg197 12885963

[53] Lepiniec L, Debeaujon I, Routaboul JM, Baudry A, Pourcel L, Nesi N, et al Genetics and biochemistry of seed flavonoids. Annu Rev Plant Biol. 2006; 57:405–30. 10.1146/annurev.arplant.57.032905.105252 16669768

[54] Ahn JH, Kim J-S, Kim S, Soh HY, Shin H, Jang H, et al De novo transcriptome analysis to identify anthocyanin biosynthesis genes responsible for tissue-specific pigmentation in zoysiagrass (*Zoysia japonica* Steud.). PLoS One. 2015; 10(4):e0124497 10.1371/journal.pone.0124497 25905914 PMC4408010

[55] Chapple C. Molecular-genetic analysis plant cytochrome P450-dependent monooxygenases. Annu Rev Plant Physiol Plant Mol Biol. 1998; 49:311–43. 10.1146/annurev.arplant.49.1.311 15012237

[56] Seitz C, Ameres S, Schlangen K, Forkmann G, Halbwirth H. Multiple evolution of flavonoid 3′,5′-hydroxylase. Planta. 2015; 242(3):561–73. 10.1007/s00425-015-2293-5 25916309

[57] Hugueney P, Provenzano S, Verries C, Ferrandino A, Meudec E, Batelli G, et al A novel cation-dependent *O*-methyltransferase involved in anthocyanin methylation in grapevine. Plant Physiol. 2009; 150(4):2057–70. 10.1104/pp.109.140376 19525322 PMC2719152

[58] Kalt W, McDonald JE, Ricker RD, Lu X. Anthocyanin content and profile within and among blueberry species. Can J Plant Sci. 1999; 79(4):617–23. 10.4141/P99-009

[59] Miosic S, Thill J, Milosevic M, Gosch C, Pober S, Molito C, et al Dihydroflavonol 4-reductase genes encode enzymes with contrasting substrate specificity and show divergent gene expression profiles in *Fragaria* species. PLoS One. 2014; 9(11):e112707 10.1371/journal.pone.0112707 25393679 PMC4231056

[60] Jaakola L. New insights into the regulation of anthocyanin biosynthesis in fruits. Trends Plant Sci. 2013; 18(9):477–83. 10.1016/j.tplants.2013.06.003 23870661

[61] Koyama R, Roberto SR, de Souza RT, Borges WFS, Anderson M, Waterhouse AL, et al Exogenous abscisic acid promotes anthocyanin biosynthesis and increased expression of flavonoid synthesis genes in *Vitis vinifera* × *Vitis labrusca* table grapes in a subtropical region. Front Plant Sci. 2018; 9:323 10.3389/fpls.2018.00323 29632542 PMC5879125

[62] Hu B, Li J, Wang D, Wang H, Qin Y, Hu G, et al Transcriptome profiling of *Litchi chinensis* pericarp in response to exogenous cytokinin and abscisic acid. Plant Growth Regul. 2018; 84(3):437–50. 10.1007/s10725-017-0351-7

